# Blood Cell Count Inflammatory Markers as Prognostic Indicators of Periodontitis: A Systematic Review and Meta-Analysis

**DOI:** 10.3390/jpm12060992

**Published:** 2022-06-17

**Authors:** Oana Almășan, Daniel-Corneliu Leucuța, Mihaela Hedeșiu

**Affiliations:** 1 Prosthetic Dentistry and Dental Materials Department, Iuliu Hațieganu University of Medicine and Pharmacy, 400006 Cluj-Napoca, Romania; oana.almasan@umfcluj.ro; 2 Department of Medical Informatics and Biostatistics, Iuliu Hațieganu University of Medicine and Pharmacy, 400349 Cluj-Napoca, Romania; 3 Department of Oral and Maxillofacial Surgery and Radiology, Iuliu Hațieganu University of Medicine and Pharmacy, 400006 Cluj-Napoca, Romania; mhedesiu@gmail.com

**Keywords:** inflammatory biomarkers, systemic inflammation, blood cells, gingival aggressive periodontitis, periodontitis prognosis

## Abstract

(1) Background: Our study aimed to assess the association between the neutrophil to lymphocyte ratio (NLR), platelet to leukocyte ratio (PLR), lymphocyte to monocyte ratio (LMR), red cell distribution width (RDW), and systemic immune inflammation index (SII) and periodontitis. (2) Methods: We searched PubMed, Embase, Scopus, Web of Science, and LILACS databases, identifying observational studies. The Newcastle Ottawa scale was used to evaluate the quality of the included studies. The principal summary outcome measure in our random effects meta-analysis was the mean difference (MD). (3) Results: After screening 682 search results, a total of 10 studies including 3164 subjects were selected for quantitative assessment. We found a higher mean NLR, PLR, and LMR in the periodontitis group compared to the control group (0.41 (95% CI 0.12–0.7), *p* = 0.006; 7.43 (95% CI 0.31–14.54), *p* = 0.04; 2.05 (95% CI 0.27–3.83), *p* = 0.024). No differences were observed for RDW. (4) Conclusions: We found an association between NLR, LMR, and PLR and periodontitis, which might be thought of as emerging blood cell count inflammatory biomarkers that could shed light on the link between periodontitis and systemic disbalances, as well as for periodontitis prognosis and grading.

## 1. Introduction

Periodontitis represents a group of chronic diseases that affect the supporting tissues of the teeth and are characterized by the destruction of periodontal tissues, the alveolar bone, and the supporting tissues of teeth. Inflammatory cells cause an immune response in the periodontal tissues [1]. The inflammatory mediators from the periodontal tissues can activate the immune system and initiate a systemic acute phase response [2]. In addition, many chronic diseases are linked to periodontitis, including cardiovascular disease, diabetes, cerebrovascular disease, neurodegenerative disease, and cancer [3]. Analyzing systemic circulatory markers, such as neutrophils [4,5], the neutrophil to lymphocyte ratio (NLR) [1], platelets [6], the platelet to leukocyte ratio (PLR) [7], and erythrocyte counts [8], could offer relevant data regarding systemic and periodontal infection.

The white blood cell count and absolute neutrophil count seem to be among the additional markers that could also help predict infection [9]. The amount of peripheral white blood cells has been reported to rise as the severity of periodontitis increases [10]. Despite the fact that these indicators are not specific to periodontitis, there may be a link between them.

In 2017, a new classification scheme for periodontitis was adopted [11]. The new periodontal disease categorization replaced the previous one [12], which uses a single term, “periodontitis”, for all earlier forms of the disease defined as “chronic” or “aggressive”.

To the best of our knowledge, there is no systematic review focusing on the relation between blood cell count inflammatory markers and periodontitis. Therefore, the objectives of this research were to carry out a systematic review and meta-analysis to investigate the association between the neutrophil to lymphocyte ratio (NLR), platelet to leukocyte ratio (PLR), lymphocyte to monocyte ratio (LMR), red cell distribution width (RDW), and systemic immune inflammation index (SII) and periodontitis.

## 2. Materials and Methods

This systematic review was performed in accordance with the recommendations of the “Preferred Reporting Items for Systematic Reviews and Meta-Analyses Protocols (PRISMA) Statement” [13].

### 2.1. Eligibility Criteria

We included in our review all the studies on periodontitis subjects assessing inflammation status using blood cell count ratios or the red cell distribution width. We excluded case reports, mechanistic articles, animal studies, reviews, editorials, and conference abstracts.

### 2.2. Information Sources

A structured electronic search was conducted in March 2022 of the following databases: PubMed, Scopus, Embase, Web of Science, and LILACS. MeSH and Emtree terms were used, where applicable. The last electronic search was performed on all databases on 15 April 2022.

### 2.3. Search Strategy

The search strategy included the terms “neutrophils”, “platelets”, “lymphocytes”, “ratio”, “systemic immune inflammation index”, “red cell distribution width”, and “periodontitis” as free text words, along with MeSH or Emtree terms (where possible), synonyms, singular as well as plural forms, and abbreviations (NLR, PLR, LMR, SII, RDW). The complete strategies adapted for each database are presented in Supplementary Table S1.

### 2.4. Selection Process

The exported lists of results from all the databases were imported in the Clarivate EndNoteTM online version [14], where the duplicates were removed by the software. Next, the remaining results were exported to an Excel spreadsheet file (Microsoft Office 365, MS, Redmond, WA, USA), which served as a selection, extraction, and quality assessment electronic form. All references were managed with Zotero software version 6.0.6 (Roy Rosenzweig Center for History and New Media, Fairfax, Virginia, USA) [15]. Two authors (A.O., L.D.C.) independently screened the titles and abstracts of all the articles manually, and, when in doubt, debated whether the paper should be considered. The selected articles were retrieved in full-text and assessed for inclusion by the same authors independently, with differences in opinion resolved by discussion. For each excluded article, the exclusion motive was recorded. For one article for which we could not locate a full-text version, we contacted the corresponding author, and he provided us with the document we needed.

### 2.5. Data Collection Process

Two reviewers extracted data from the articles in the standardized Excel form file.

### 2.6. Data Items

Data were extracted using a standardized form, which included the following information: (1) author names and year of publication; (2) title; (3) abstract; (4) publication title; (5) keywords; (6) study selection; (7) screening; (8) inclusion criteria; (9) exclusion criteria; (10) population; (11) exposure; (12) age; (13) gender; (14) other characteristics; (15) outcomes (NLR, PLR, LMR, RDW), i.e., mean and standard deviations; and (16) studies’ quality assessment.

### 2.7. Study Risk of Bias Assessment

All the selected articles were independently assessed regarding their methodological quality by two reviewers, and differences in assessment were resolved by discussion. The Newcastle Ottawa Scale [16] for case-control studies was used to identify the sources of bias.

### 2.8. Effect Measures

For all the outcomes, the mean difference was used as an effect measure, along with its confidence interval.

### 2.9. Synthesis Methods

If we could not identify the mean and standard deviation for the outcomes we were interested in and only found medians, we used the formula supplied by Hozo SP et al. to convert the range and number of participants [17]. Although some papers lacked the needed numbers, we were able to identify them in their charts. We extracted the numerical values from the figures using WebPlotDigitizer [18].

All meta-analyses were performed with the random effects model using the method of restricted maximum likelihood to estimate the heterogeneity variance, as we anticipated a clinical heterogenicity between studies. The Χ2-based Q-test and I^2^ were used to assess between-study heterogeneity, according to the Cochrane Handbook’s recommendations [19]

Meta-analyses were carried out in the R environment for statistical computing and graphics, version 4.1.2 [20], using the meta R package [21]. The leave-one-out sensitivity analyses, performed in case of important heterogeneity meta-analyses, as well as meta-analysis diagnostics to identify influential studies were carried out with the dmetar package [22]. No subgroup analyses were performed.

### 2.10. Reporting Bias Assessment

Since the number of selected studies was low, publication bias assessment is underpowered. Nevertheless, we computed the Egger test and plotted funnel plots to assess the presence of publication bias.

## 3. Results

### 3.1. Study Selection

**Figure 1:**
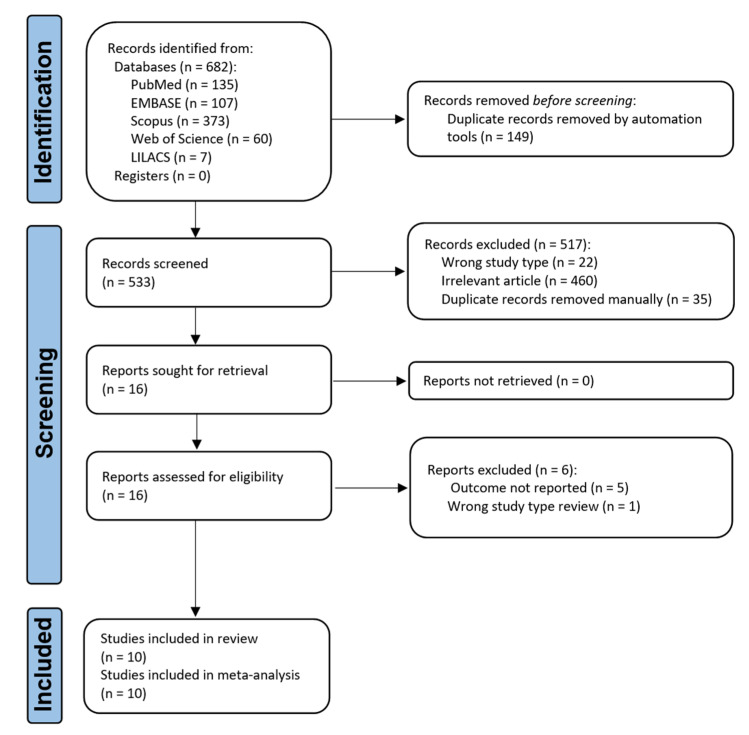
Flow diagram of the literature search and selection criteria adapted for PRISMA 2020.

A description of the search process and selection is presented in a PRISMA flow diagram (Figure 1). A total of 682 publications were found in the initial search (PubMed *n* = 135, EMBASE *n* = 107, Scopus *n* = 373, Web of Science *n* = 60, LILACS *n* = 7). After being identified as duplicates, a total of 149 studies were deleted. Following the removal of duplicates, 533 articles were subjected to a preliminary screening that included a review of the title and abstract for compliance with the inclusion and exclusion criteria. Then, 110 articles were eliminated during the screening process. There were 460 irrelevant articles and 22 wrong study types, and 35 duplicate records. We looked for the complete text of 16 articles, and for one article, we contacted the authors through email, and finally, we managed to collect all the necessary files. We read and evaluated the whole text thoroughly in order to determine the remaining articles’ eligibility. Six of these articles were eliminated for the following reasons: outcome not reported (*n* = 5) or wrong study type, i.e., review (*n* = 1). As a result, the qualitative and quantitative synthesis included 10 articles.

### 3.2. Study Characteristics

Table 1 and Supplementary Table S2 summarizes the basic characteristics of the studies that were included. A total of 3164 subjects were included in this systematic review and meta-analysis.

Eight studies used a cross-sectional study design, and two used a case-control study design. All investigations were conducted in Asia (Turkey *n* = 4, India *n* = 4, Thailand *n* = 1, China *n* = 1). Two articles assessed gingival aggressive periodontitis and eight articles observed chronic periodontitis as a case group; all articles compared them to healthy controls. The periodontitis classification differed between articles, with seven using World Workshop on the Classification of Periodontal and Peri-Implant Diseases and Conditions (1999 or 2017 versions); one used the Centers for Disease Control and Prevention, American Academy of Periodontology classification; and two did not stipulate any classification system. Six studies had a mean age of subjects between 26 and 40, three studies had a mean age of around 50, and one study did not report the age range. Most studies reported a similar distribution of gender between the groups. Concerning outcomes, seven studies reported NLR, four reported PLR and RDW, one reported LMR, and no article was identified to report SII.

### 3.3. Results of Syntheses

#### 3.3.1. Neutrophil to Lymphocyte Ratio

**Figure 2:**
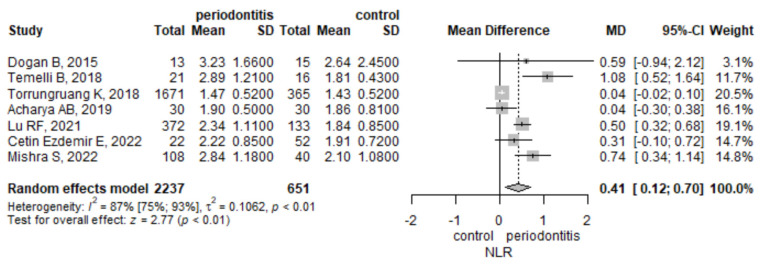
Neutrophile to lymphocyte ratio-mean difference between periodontitis and control subjects.

The meta-analysis of seven studies found that the mean NLR was higher by 0.41 (95% CI 0.12–0.7), *p* = 0.006, in the periodontitis group compared to the control group (Figure 2). There was a statistically significant heterogeneity, measured with I^2^ = 86.8% (95% CI 74.9–93%), *p* = < 0.001. To assess the robustness of the result, we performed a leave-one-out sensitivity analysis (Supplementary Figure S1). The result remained statistically significant after excluding each of the articles included in the meta-analysis. The influence analysis indicated the Torrungruang [29] study to be influential. When removing the Torrungruang study [29], the heterogeneity was the lowest, dropping to 62%, as assessed by I^2^, while with the removal of any other study, the heterogeneity was greater than or equal to 81%.

#### 3.3.2. Platelet to Leucocyte Ratio

**Figure 3:**
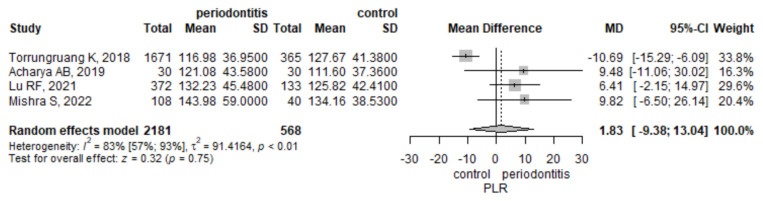
Platelet to leucocyte ratio-mean difference between periodontitis and control subjects.

Four studies analyzed the values of PLR. The meta-analysis found a mean PLR higher by 1.83 (95% CI −9.38–13.04) in the periodontitis group compared to the control group, but the result was not statistically significant, *p* = 0.749 (Figure 3). There was a statistically significant heterogeneity, measured with I^2^ = 83.1% (95% CI 56.9–93.4%), *p* = < 0.001. To assess the robustness of the result, we performed a leave-one-out sensitivity analysis (Supplementary Figure S2). The influence analysis indicated the Torrungruang study [29] to be influential. When removing the Torrungruang study [29], the result became statistically significant, and the heterogeneity dropped to 0%, as assessed by I^2^. When removing any other study, the pooled estimate did not reach statistical significance.

#### 3.3.3. Lymphocyte to Monocyte Ratio

**Figure 4:**
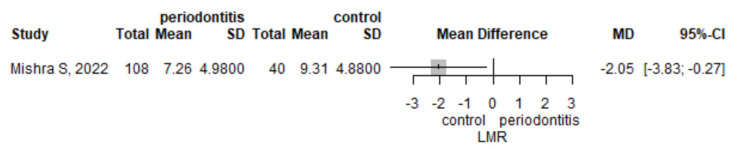
Lymphocyte to monocyte ratio -mean difference between periodontitis and control subjects.

The mean LMR was lower in the periodontitis group compared to the control group (mean difference of 2.05 (95% CI 0.27–3.83), *p* = 0.024, as observed in one study, Mishra et al. [26]) (Figure 4).

#### 3.3.4. Red Cell Distribution Width

**Figure 5:**
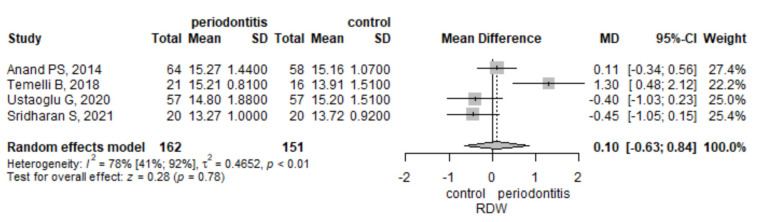
Red cell distribution width-mean difference between periodontitis and control subjects.

The meta-analysis of four studies that assessed RDW observed that its mean values were higher by 0.1 (95% CI −0.63–0.84) in the periodontitis group compared to the control group, but the result was not significant, *p* = 0.782 (Figure 5). There was a statistically significant heterogeneity, measured with I^2^ = 78.1% (95% CI 40.9–91.9%), *p* = 0.003. To assess the robustness of the result, we performed a leave-one-out sensitivity analysis (Supplementary Figure S3). The influence analysis indicated the Temelli B et al. study [28] to be influential. When removing the Temelli study [28], the result did not reach the significance level, although the heterogeneity dropped to 30%. In the case of any other study removal, the heterogeneity was greater than or equal to 81%.

### 3.4. Risk of Bias in Studies

We used the Newcastle Ottawa scale (NOS) [16] to assess the methodological quality of the included studies (Table 2). For the objective of our review, we used the case-control subsection of the NOS.

Regarding the selection section of the NOS scale, all the studies used pre-defined, transparent criteria to identify the presence of the disease (chronic periodontitis or aggressive periodontitis) and controls; thus, the case and control definitions were adequate. Nevertheless, the authors of the studies used different criteria to identify the disease according to official classifications (Periodontal Disease Classification, American Association of Periodontology 1999-three studies [23,25,28]; Classification of Periodontal and Peri-Implant Diseases and Conditions 2017-four studies [1,2,26,27]; Center of Disease Control and Prevention, American Academy of Periodontology Periodontal Disease Classification CDC/AAP-one study [29]), or no specified classification [7,24]. Only two studies (20%) out of ten had clear representativeness of the cases [23,28]. Two studies had a poor selection of controls [7,27], and two studies had a correct selection of controls. For the other six, it might be difficult to assess if the selection of controls was correct. Half of the studies enrolled subjects from the department of periodontology [1,2,7,23,26]; the other studies enrolled subjects from the internal medicine [28] or cardiology departments [24], or from an electrical company [29], and in two studies, the population was not stated [25,27].

Concerning comparability, only one study used matching for age and gender [27], and all the other studies performed a simple comparison between cases and controls. Nevertheless, all the studies used extensive exclusion criteria for systemic diseases, which help in group comparability. A problem in comparability might be the smoking status, as four studies had a percentage under 25% of smokers or occasional smokers [25,27,28].

All research can be regarded as bias-free when it comes to assessing blood parameters since the laboratory methods used were reliable, the same methods for cases and controls were used, and there was no non-response rate.

The biggest issue in the methodology of these studies is the representativeness of the cases. The possible problem with control selection, as well as the comparability of the groups, appears to be less affected.

### 3.5. Reporting Biases

Since the number of studies in each meta-analysis was below ten, publication bias could not be reliably assessed. Nevertheless, the *p*-values for the Egger test for assessing publication bias were greater than the level of significance.

## 4. Discussion

This is the first systematic review and meta-analysis, to our knowledge, investigating the association between blood cell count inflammatory biomarkers and periodontitis. Periodontitis was found to have a statistically significant relationship with NLR and LMR, a debatable association with PLR, and RDW did not approach the significance level.

The neutrophil to lymphocyte ratio and platelet to lymphocyte ratio are two biomarkers of systemic inflammation that have increasingly gained interest in the diagnosis of a variety of cardiovascular diseases [30,31,32], diabetes [33,34,35], inflammatory diseases [36,37], and malignancies [38,39]. In our review, we found higher mean values of NLR in periodontitis patients compared to healthy controls. Although there was an important heterogeneity, the result was robust even when performing the leave-one-out sensitivity analyses. Furthermore, a prospective study by Acharya et al. [7] objectivated the reduction of NLR and PLR levels after scaling and root planing.

Concerning PLR, the initial analysis did not reach statistical significance, but after the removal of the statistically influential study of Torrungruang et al. [29], the result became statistically significant and without heterogeneity. Twenty-five percent of the subjects in this study were with impaired glucose tolerance or diabetes, while all the other studies that reported PLR were free of systemic diseases; thus, this exclusion is likely to be warranted.

Little is known-and remains unknown-about the association between LMR and periodontitis, which could serve as a possible marker of systemic inflammation as well as aid in diagnosing and predicting the prognosis of periodontitis. Only one study reported the association between periodontitis and LMR, and it proved to be statistically significant, with higher values being observed in the periodontitis group.

Several studies found a link between periodontal disorders and low erythrocyte count, implying that periodontal diseases could be linked to chronic anemia [40,41]. RDW has also been linked to inflammatory markers, including erythrocyte sedimentation rate and high-sensitivity C-reactive protein, which have both been linked to periodontitis [42]. In our meta-analysis, we could not objectivate a statistically significant association between RDW and periodontitis, the results being heterogeneous, with studies showing conflicting results concerning the direction of the association.

Leite et al., in a systematic review and meta-regression, studied the effect of smoking on periodontitis and showed that periodontitis is aggravated by smoking [43]. Larvin et al. used a unique artificial intelligence-based network analysis to identify systemic multimorbidity clusters in subjects with periodontitis and analyzed factors that may influence the severity of those clusters. The authors stated that hypertension, arthritis, and obesity had the largest impact on multimorbidity clusters in subjects with periodontitis, and diabetes was more prevalent in those who had experienced a greater degree of clinical attachment loss. they also showed that in adults with severe periodontitis, smoking status influenced the clustering pattern of diabetes and cancer [44]. The authors of the chosen studies from our systematic review and meta-analysis evaluated the smoking status in different ways: the authors Acharya AB et al. [7] and Lu RF et al. [25] did not discuss smoking. Çetin Özdemir E et al. [1], Mishra S et al. [26], and Ustaoglu G et al. [2] studied non-smoking subjects; thus smoking was excluded as a confounder. In the study of Dogan B et al. [24], almost the entire population consisted of non-smokers, and therefore, the results were interpreted as independent of the effects of smoking. The authors Anand PS et al. [23] discussed smoking, and they performed a logistic regression model adjusted for age, gender, smoking, and body mass index. Sridharan S et al. [27] and Temelli B at al. [28] discussed smoking and found no significant differences between smokers and non-smokers. Torrungruang K et al. [29] discussed the smoking status and did not show the relation between periodontitis and the biomarkers of interest.

Michaud et al., in a review, showed that periodontitis is common in adults, and it worsens with age [45]. Ciesielska et al., in a narrative review, showed that menopausal women have a higher risk of periodontal disease [46]. In the publications selected in our review and meta-analysis, the authors Çetin Özdemir et al. [1] showed that the mean age of the individuals in the periodontitis group was higher than the other groups, and gender was significantly different. Dogan B et al. revealed a higher proportion of females in the risk factor groups and a higher age [24]. Sridharan S et al. showed that age was significantly related to red cell distribution width, but not gender [27]. Temelli B et al. found only age differences among groups [28]. No significant differences in age and gender distribution between the groups were found by Lu RF et al. [25], Mishra S et al. [26], Torrungruang K et al. [29], or Ustaoglu G et al. [2].

Our pooled results ring a bell, paving the way for future studies that could identify viable strategies for evaluating blood cell count inflammatory biomarkers for diagnostic, prognostic, and therapeutic management. Moreover, this study emphasizes that periodontitis has a repercussive potential upon systemic inflammation, being a response to bacterial infection.

### 4.1. Limitations and Strengths

There are several limitations of our study as well as of the included studies. The observational nature of the studies precludes any cause and effect relationship affirmations. This is further limited by the fact many of the studies were cross-sectional, a design that cannot assess the directional relation between the inflammatory biomarkers and periodontitis. Only one study assessed the effects of periodontal treatment on NLR and PLR that significantly lowered their values. There was a high heterogeneity between the results of the studies. To check the robustness of our results, we performed leave-one-out sensitivity analyses, and NLR results resisted. Furthermore, PLR became statistically significant after the removal of one study that had other comorbidities, and the heterogeneity dropped to 0. Nevertheless, for RDW, the heterogeneity remained important. For several of the outcomes, we found only a few studies to support our meta-analyses since the literature is still emerging on this topic. Concerning the methodological quality, the selected studies had two more relevant drawbacks: the representativeness of the cases was problematic since many studies did not report if all the cases in the accessible population were included in their research, and the reporting of the selection of controls, whether derived from the same population as the cases, was lacking in several studies. Nevertheless, the other quality criteria were fulfilled. Although the majority of the studies reported the classification system used to diagnose periodontitis, there was some heterogeneity between them regarding the type or the year of classification. Nevertheless, the systems used are from trusted authorities on the subject.

### 4.2. Study Strengths

The study’s key strength is that it primarily assessed four commonly viable non-invasive biomarkers that may be easily used in a clinical context. To the best of our knowledge, this is the first systematic review and meta-analysis on this emerging topic. Our search technique was thorough and included many medical databases, allowing us to explore the link in a systematic manner across a wide range of populations, resulting in more generalizable findings. Furthermore, the review has a robust methodology and uses a solid approach that includes sensitivity analyses and quality assessments of the selected publications.

### 4.3. Implications for Practice and Future Research

These inflammatory markers could be included as potential parameters to assess the impact of periodontitis on systemic health, adding to the burden of other inflammation-generating diseases. Furthermore, they could be used as prognostic markers for the evolution of the disease and the treatment efficiency assessment on a systemic level. Moreover, it would be possible to employ the inflammatory markers in a way that uses standardized values to allow for consistent periodontal disease diagnosis and severity grading. Through the creation and utilization of biological instruments to make a diagnosis and evaluate treatment outcomes, this method will serve as a key step toward the medicalization of periodontics and dentistry [47].

To gain evidence in these directions, future prospective designed studies should be endeavored.

If their role is confirmed, clinicians could better classify and monitor the disease progression and treatment efficiency with these inflammatory markers.

## 5. Conclusions

Our systematic review and meta-analysis found an association between NLR, LMR, and PLR and periodontitis, but not for RDW. Thus, these ratios might be thought of as emerging blood cell count inflammatory biomarkers that could shed light on the link between periodontitis and systemic disbalances, as well as for periodontitis prognosis and grading. However, because the methodological quality of the evaluated research is imperfect, the results should be used with care. Further prospective design studies are warranted.

## Figures and Tables

**Table 1:** Characteristics of the included studies.

Author, Year of Publication	Country	Region	Study Design	Study Population	Age (Years): Mean ± (SD) /(Range) Case vs. Control	Female (%) Case vs. Control	Outcome Parameters	Periodontitis Classification
Acharya AB, 2019 [7]	India	Asia	PC	CP vs. H	39.6 ± 0.96 vs. 39.6 ± 0.96	50% vs. 50%	NLR, PLR	NCS
Anand PS, 2014 [23]	India	Asia	CS	GAP vs. H	32.80 ± 7.21 vs. 30.40 ± 7.60	50% vs. 43%	RDW	ICWP 1999
Çetin Özdemir E, 2022 [1]	Turkey	Asia	CS	CP vs. H	38.36 ± 7.02 vs. 35.3 ± 9.88	36% vs. 73%	NLR	WWC 2017
Dogan B, 2015 [24]	Turkey	Asia	CS	CP vs. H	NR	32%	NLR	NCS
Lu RF, 2021 [25]	China	Asia	CC	GAP vs. H	27.50 ± 5.24 vs. 26.77 ± 5.05	59% vs. 60%	NLR, PLR	WWC 1999
Mishra S, 2022 [26]	India	Asia	CC	CP vs. H	30.67 ± 4.89 vs. 30.67 ± 4.89	45% vs. 48%	NLR, PLR, LMR	WWC 2017
Sridharan S, 2021 [27]	India	Asia	CS	CP vs. H	50.8 ± 10 vs. 41.6 ± 3.4	65% vs. 65%	RDW	WWC 2017
Temelli B, 2018 [28]	Turkey	Asia	CS	CP vs. H	50 (42–71) vs. 49 (33–65)	33% vs. 63%	NLR, RDW	WWC 1999
Torrungruang K, 2018 [29]	Thailand	Asia	CS	CP vs. H	48.0 ± 5.0	28%	NLR, PLR	CDC/AAP 2007
Ustaoglu G, 2020 [2]	Turkey	Asia	CS	CP vs. H	37.4 ± 7.0 vs. 35.6 ± 7.0	44% vs. 54.3%	RDW	WWC 2017

PC-prospective cohort; CS-cross-sectional; CC-case-control; CP-chronic periodontitis; GAP-gingival aggressive periodontitis; H-healthy; ICWP-International Workshop for Classification of Periodontal Disease and Conditions; WWC-World Workshop on the Classification of Periodontal and Peri-Implant Diseases and Conditions; CDC/AAP-Centers for Disease Control and Prevention (CDC), American Academy of Periodontology (AAP); NLR-neutrophil to lymphocyte ratio; PLR-platelet to leukocyte ratio; LMR-lymphocyte to monocyte ratio; RDW-red cell distribution width; NR-not reported; NCS-no classification system.

**Table 2:** Newcastle Ottawa Scale quality assessment of the selected articles.

Author and Year of Publication	Cases DA	Cases R	Controls S	Controls D	Cases and Controls C ^†^	EA	Cases and Controls A ^††^	NRR
Acharya AB, 2019 [7]	*			*	^#^	*	*	*
Anand PS, 2014 [23]	*	*	?	*	^#^	*	*	*
Çetin Özdemir E, 2022 [1]	*		?	*	^#^	*	*	*
Dogan B, 2015 [24]	*		*	*	^#^	*	*	*
Lu RF, 2021 [25]	*		?	*	^#^	*	*	*
Mishra S, 2022 [26]	*		?	*	^#^	*	*	*
Sridharan S, 2021 [27]	*			*	^ (age, gender)	*	*	*
Temelli, B, 2018 [28]	*	*	*	*	^#^	*	*	*
Torrungruang K, 2018 [29]	*		?	*	^#^	*	*	*
Ustaoglu G, 2020 [2]	*		?	*	^#^	*	*	*

DA-definition adequacy; R-representativeness; D-definition; EA-exposure ascertainment; A-ascertainment; ^†^-according to design or analysis; ^††^-same method; ^-matched for the variables in the brackets; NRR-non-response rate; *-fulfilled criteria; ?-unclear criteria; ^#^-extensive exclusion criteria.

## Data Availability

Data is contained within the article.

## Supplementary Materials

The following supporting information can be downloaded at: https://www.mdpi.com/article/10.3390/jpm12060992/s1, Figure S1: Leave-one-out sensitivity analysis for neutrophils to lymphocyte ratio; Figure S2: Leave-one-out sensitivity analysis for platelet to leucocyte ratio; Figure S3: Leave-one-out sensitivity analysis for red cell distribution width; Table S1: Search strategies for each database; Table S2: Inclusion and exclusion criteria of the subjects within the original articles.
