# Supplementary material for: Blood Cell Count Inflammatory Markers as Prognostic Indicators of Periodontitis: A Systematic Review and Meta-Analysis
Source: J Pers Med. 2022 Jun 17;12(6):992. doi: 10.3390/jpm12060992 (PMC9225277; doi:10.3390/jpm12060992)
Supplement: Supplementary file 1 [file jpm-12-00992-s001.zip › jpm-1737736-supplementary.pdf]

Journal of *Personalized Medicine*

MDPI

Supplementary File

Blood cell count inflammatory markers and periodontitis: A systematic review and meta-analysis

Oana Almășan, Daniel-Corneliu Leucuța and Mihaela Hedeșiu

Funding: This work was granted by project PDI-PFE-CDI 2021, entitled "Increasing the Performance of Scientific Research, Supporting Excellence in Medical Research and Innovation, PROGRES", no. 40PFE/30.12.2021 of Iuliu Hațieganu University of Medicine and Pharmacy, Cluj-Napoca, Romania.

Table S1. Search strategies for each database.

PubMed
((((("neutrophils"[MeSH Terms] OR "neutrophil"[All Fields] OR "neutrophile"[All Fields] OR "neutrophiles"[All Fields] OR "neutrophils"[All Fields] OR "Polymorphonuclear"[All Fields] OR "granulocyte"[All Fields] OR "pmn"[TIAB] OR "pmn"[OT]) OR ("Blood Platelets"[Mesh] OR "Platelet Count"[Mesh] OR "platelet"[All Fields] OR "platelets"[All Fields] OR "Thrombocyte"[All Fields] OR "Thrombocytes"[All Fields]) OR ("Monocytes"[Mesh] OR "Monocyte"[All Fields] OR "Monocytes"[All Fields])) AND ("lymphocytes"[MeSH Terms] OR "lymphocyte count"[MeSH Terms] OR "lymphocyte"[All Fields] OR "lymphocytes"[All Fields] OR "lymphocyts"[All Fields]) AND ("ratio"[All Fields] OR "ratios"[All Fields])) OR "PLR"[All Fields] OR "NLR"[All Fields] OR "LMR"[All Fields] OR "Systemic immune-inflammation index"[All Fields] OR "SII"[All Fields]) OR ("red cell distribution width"[All Fields] OR RDW[Title/Abstract])) AND ("periodontitis"[MeSH Terms] OR "periodontitis"[All Fields] OR "parodontitis"[All Fields] OR "paradontitis"[All Fields])
**EMBASE**
((((('neutrophil'/exp OR 'neutrophil count'/exp OR 'neutrophil' OR 'neutrophile' OR 'neutrophiles' OR 'neutrophils' OR 'Polymorphonuclear' OR 'granulocyte' OR 'pmn' OR 'pmn') OR ('thrombocyte'/exp 'Blood Platelets' OR 'Platelet Count'/exp OR 'platelet' OR 'platelets' OR 'Thrombocyte' OR 'Thrombocytes') OR ('monocyte'/exp OR 'monocyte count'/exp OR 'monocyte' OR 'monocytes')) AND ('lymphocyte'/exp OR 'lymphocyte count'/exp OR 'lymphocyte' OR 'lymphocytes' OR 'lymphocyts') AND ('ratio' OR 'ratios')) OR 'PLR' OR 'NLR' OR 'LMR' OR 'neutrophil lymphocyte ratio'/exp OR 'platelet lymphocyte ratio'/exp OR 'monocyte lymphocyte ratio'/exp OR 'systemic immune inflammation index'/exp OR 'Systemic immune-inflammation index' OR 'SII') OR ('red cell distribution width'/exp OR 'red cell distribution width' OR RDW)) AND ('periodontitis'/exp OR 'periodontitis' OR 'parodontitis' OR 'paradontitis')
**Scopus**
TS=((((((neutrophil OR neutrophile OR neutrophiles OR neutrophils OR polymorphonuclear OR granulocyte OR pmn) OR (blood AND platelets OR platelet OR platelets OR thrombocyte OR thrombocytes) OR (monocyte OR monocytes)) AND (lymphocyte OR lymphocytes OR lymphocyts) AND (ratio OR ratios)) OR plr OR nlr OR lmr OR "neutrophil lymphocyte ratio" OR "platelet lymphocyte ratio" OR "monocyte lymphocyte ratio" OR "systemic immune inflammation index" OR "Systemic immune-inflammation index" OR sii) OR ("red cell distribution width" OR rdw)) AND (periodontitis OR parodontitis OR paradontitis))
**Web of Science**
TS=(("irritable bowel syndrome" OR ("irritable" AND "bowel" AND "syndrome") OR ("Colon" AND "Irritable") OR "IBS") AND (("meditation" OR "meditations" OR "meditation's" OR "meditational" OR "meditative" OR "meditator" OR "meditators" OR "meditate" OR "meditated" OR "meditating" OR "spiritual therapy" OR "spiritual healing" OR "prayer" OR "tai chi" OR "qi gong") OR ("mindfulness" OR "mindful" OR "Self-Compassion" OR "MBT" OR "MBCT" OR "MBSR") OR ("Mind-Body Therapies" OR "mind-body" OR "mind body" OR "mental healing" OR "faith healing" OR "yoga"))) Filter = Article
**LILACS**
tw:((((((neutrophil OR neutrophile OR neutrophiles OR neutrophils OR polymorphonuclear OR granulocyte OR pmn) OR (blood AND platelets OR platelet OR platelets OR thrombocyte OR thrombocytes) OR (monocyte OR monocytes)) AND (lymphocyte OR lymphocytes OR lymphocyts) AND (ratio OR ratios)) OR plr OR nlr OR lmr OR "neutrophil lymphocyte ratio" OR "platelet lymphocyte ratio" OR "monocyte lymphocyte ratio" OR "systemic immune inflammation index" OR "Systemic immune-inflammation index" OR sii) OR ("red cell distribution width" OR rdw)) AND (periodontitis OR parodontitis OR paradontitis))

Table S2. Inclusion and exclusion criteria of the subjects within the original articles.

Author, year of publication	Inclusion criteria	Exclusion criteria
**Acharya AB, 2019 [7]**	CP defined as the presence of at least 20 natural teeth with generalized (i.e., >30% of the sites examined) probing pocket depths (PPD) of = 4 mm and clinical attachment level (CAL) of = 2 mm (stent as reference); positive for bleeding on probing; radiographic evidence of bone loss.	Patients with systemic diseases; known allergies; tobacco users.
**Anand PS, 2014 [23]**	Patients were diagnosed as having GAP based on the criteria proposed by the International Workshop for Classification of Periodontal Diseases and Conditions in 1999 and were included in the study if they had a probing depth and a clinical attachment level of ≥5 mm on at least eight permanent teeth, of which at least three were not permanent first molars or incisors. For the control group, participants were considered as periodontally healthy if they had no site with probing depth and clinical attachment level of >3 mm and if <20% of sites exhibited bleeding on probing.	Pregnant women, individuals with a history of any systemic diseases, or a history of any antibiotic therapy or periodontal treatment during the 12 months before the examination.
**Çetin Özdemir E, 2022 [1]**	74 systemically healthy, non-smoking individuals. Chronic diseases such as periodontitis. Group 3 included 22 patients with generalized stage 3 periodontitis, with interdental CAL ≥5 mm, PD ≥6 mm, and radiographic bone loss extending to the middle of the root or beyond.	Any systemic disease, smokers, pregnant or breastfeeding, any antibiotic or anti-inflammatory drugs, received any periodontal treatment within the past 6 months.
**Dogan B, 2015 [24]**	Participants were classified as periodontitis (P) or non-periodontitis (NP). The groups were assigned as follows: NP group, individuals with teeth having PD <5 mm; P group, individuals with ^{3}1 tooth with PD ^{3}5 mm and CAL ^{3}4 mm.	Pregnancy or lactating at the time of the study; history of chemotherapy, radiotherapy, or renal diseases; history of systemic antibiotic administration within the previous 3 months and hormone replacement therapy; history of periodontal treatment within the past 6 months. The control group did not have diabetes, hyperlipidemia, obesity, or post-menopausal syndrome.
**Lu RF, 2021 [25]**	The inclusion criteria for the GAgP group were based on the 1999 International Workshop for the Classification of Periodontal Diseases and Conditions1: (i) 16 to 35 years old; (ii) presented with at least 20 functional teeth in the mouth; (iii) probing depth (PD) >5 mm and attachment loss (AL) >3 mm in over six teeth, with radiographic evidence of alveolar bone loss, and at least three of the affected teeth were not incisors or first molars. Healthy controls were recruited from volunteers	Exclusion criteria of participants were (i) pregnancy; (ii) lactation period; (iii) intake of antibiotics or anti-inflammatory drugs in the past 3 months; (iv) systemic diseases; (v) history of periodontal treatment within 6 months; or (vi) history of orthodontic treatment. All smokers were excluded from the study to avoid potential confounding variables.

	or staff and students†. Inclusion criteria were (i) age below 36, (ii) teeth with PD ≤3 mm, and (iii) no clinical evidence of AL.	
**Mishra S, 2022 [26]**	Categorization of subjects into case and control groups was based on the classification criteria proposed by the 2017 World Workshop Classification System for Periodontal and Peri-Implant Diseases and Conditions [17]. The case group comprised systemically healthy subjects with generalized stage 3 grade C periodontitis while the control group consisted of participants who were systemically healthy and also exhibited periodontal health.	Patients with history of use of tobacco in any form, diabetes mellitus, cardiovascular diseases, renal, hematological, inflammatory or any other known systemic disorders or those under any medication including anti-inflammatory drugs and nutritional supplements; pregnant and lactating women were excluded from the present study. Subjects who had undergone any periodontal therapy within the past 6 months were also excluded from the study.
**Sridharan S, 2021 [27]**	Eighty gender-matched individuals aged between 40 and 60 years with a minimum of 20 teeth (excluding third molars) were enrolled in this study. Chronic periodontitis.	Individuals were excluded from the study if they had any of the following: obesity (BMI >30) according to the World Health Organization, 1995; any history of blood dyscrasia such as anemia and thrombocytopenia; systemic diseases such as diabetes mellitus and thyroid disease; renal disorders; cardiac disorders (valve problems congenital heart disease); taking any drug which could affect the blood pressure apart from antihypertensive drugs; smokers, both former and current, showing high dependency according to the Fagerstrom test score; pregnant women. None of the included individuals had received treatment for periodontal disease within 6 months before commencement of the study. Occasional smokers (showing low dependency on the Fagerstrom test score) were included, although in minimal numbers (n = <10%).
**Temelli B, 2018 [28]**	The periodontal diagnosis of the patients (having periodontitis or not) was made according to the criteria proposed by the 1999 International Workshop for the Classification of Periodontal Disease and Conditions.	A diagnosis of and/or being treated for CAD, having diabetes mellitus, using statins, using calcium channel blockers for hypertension therapy, pregnancy, lactation, being under the age of 30, having rheumatologic disease or malignant disease, used/using anti-inflammatory and/or antibiotics in the past 3 months/currently, and having undergone periodontal therapy within 6 months.
**Torrungruang K, 2018 [29]**	The participants were divided into three groups: normoglycemia, impaired fasting glucose (IFG), and diabetes. The periodontal status of each participant was determined using both categorical and continuous variables. In accordance with the Centers of Disease Control and Prevention and American Academy of	Individuals with at least six teeth and complete data on all variables were included for analysis. Individuals with systemic conditions potentially affecting serum inflammatory biomarkers (rheumatoid arthritis, systemic lupus erythematosus, psoriasis, or malignancy) were excluded.

	Periodontology (CDC/AAP) case definitions, the participants were categorized into three groups: no/mild, moderate, or severe periodontitis.	
**Ustaoglu G, 2020 [2]**	The study participants comprised 57 patients (28 patients were diagnosed with stage 3 periodontitis or were determined to be periodontally healthy based on the criteria proposed by the International Workshop for Classification of Periodontal Diseases and Conditions in 2017. Patients were included in the study if they were systemically healthy and found to have a probing depth (PD) of ≥6 mm and interdental clinical attachment level (CAL) of ≥5 mm, if they had tooth loss of ≤4 teeth due to periodontitis, and if they had radiographically detected bone loss reaching the mid-third of the root and beyond. Periodontally healthy subjects were included in the study if they had no radiographically detected bone loss and if they had no sites with clinical attachment loss and no sites with probing depth (PD) of >3 mm in their oral cavity.	The age range of the participants was between 18 and 50 years. Since individuals older than 50 years might have already developed atherosclerotic processes or might have comorbid diseases, they were excluded from the study as these potential conditions could interfere with the results of the complete blood count tests. Individuals were excluded if they had a history of cardiovascular disease, diabetes mellitus, hypertension, upper respiratory tract infections, smoking, hypo/hyperthyroidism, chronic renal failure, malignancy, any hematological abnormalities, or any medication use such as antiplatelet agents, anticoagulants, antihyperlipidemic, angiotensin-converting enzyme inhibitors, and steroids. Individuals who had been treated for periodontitis in the past 6 months were also excluded.

Sensitivity analysis

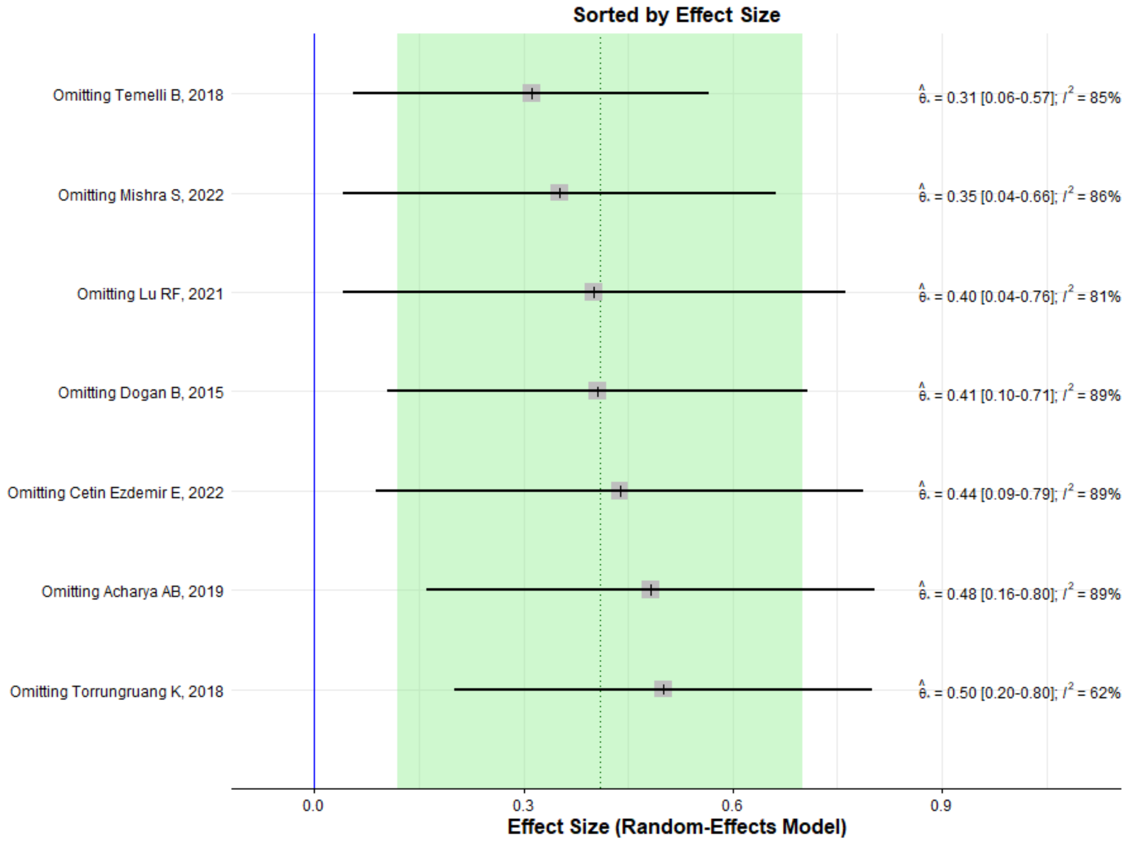

Supplementary Figure S1. Leave-one-out sensitivity analysis for neutrophils to lymphocyte ratio. $\hat{\theta}$ = neutrophyle to lymphocyte ratio mean difference.

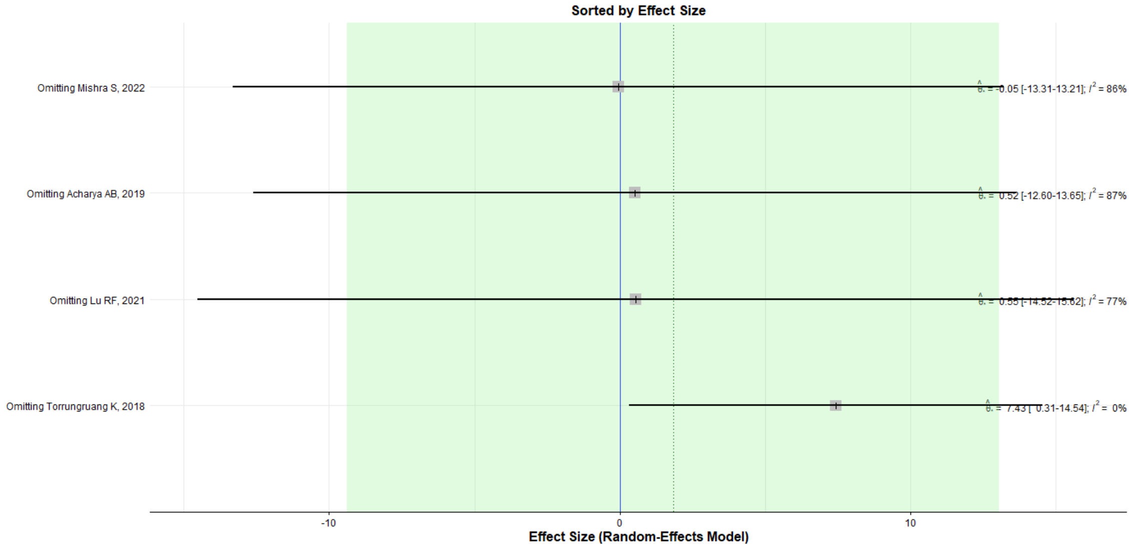

Supplementary Figure S2. Leave-one-out sensitivity analysis for platelet to leucocyte ratio. $\hat{\theta}$ = platelet to leucocyte ratio mean difference.

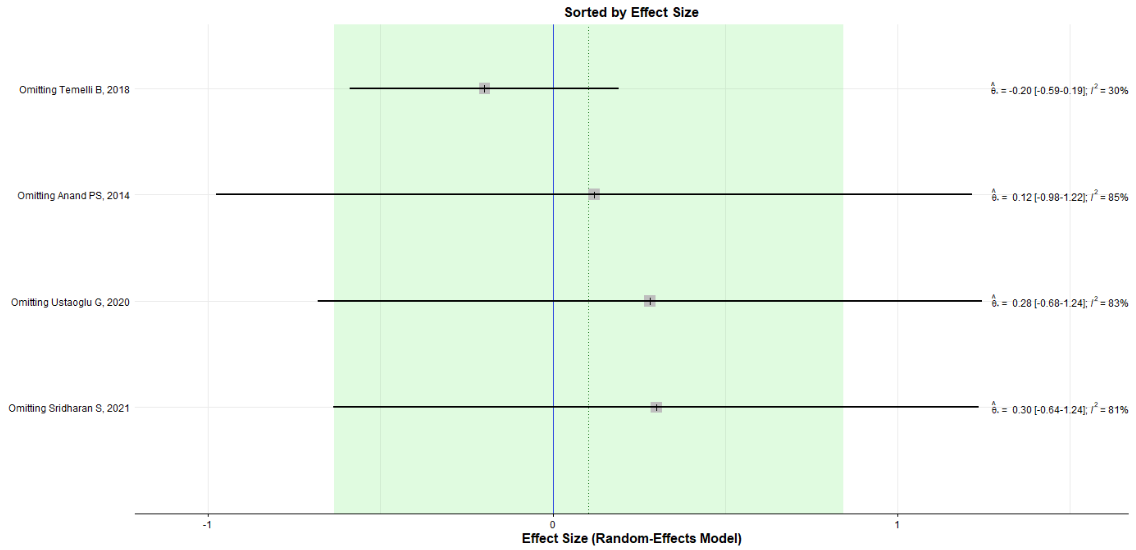

Supplementary Figure S3. Leave-one-out sensitivity analysis for red cell distribution width. $\hat{\theta}$ = red cell distribution width mean difference.
